# Letter-to-the-editor to the study by Bartek J *et al* “Multidisciplinary consensus-based statement on the current role of Middle Meningeal Artery embolization (MMAE) in chronic SubDural Hematoma (cSDH)” – A letter from the international CSDH research group committee, iCORIC

**DOI:** 10.1016/j.bas.2024.104169

**Published:** 2024-12-24

**Authors:** T.S.R. Jensen, J. Duerinck, D. Holl, C. Iorio-Morin, J. Soleman, E. Edlmann, iCORIC steering committee

**Affiliations:** a Department of Neurosurgery, Copenhagen University Hospital, Copenhagen, Denmark; b Department of Neurosurgery, Universitair Ziekenhuis Brussel, Vrije Universiteit Brussel, Brussels, Belgium; c Department of Neurosurgery, Erasmus Medical Center, Erasmus MC Stroke Center, Rotterdam, the Netherlands; d Division of Neurosurgery, Department of Surgery, Centre Hospitalier Universitaire de Sherbrooke, Sherbrooke, Canada; e Department of Neurosurgery, University Hospital Basel, Basel, Switzerland; f Department of Neurosurgery, South West Neurosurgical Centre, Plymouth, United Kingdom

Dear Editor,

We have read with great interest the consensus statement on middle meningeal artery embolization (MMAE) in chronic subdural hematoma (CSDH) by Bartek J *et al* (Bartek et al., 2024). In a multidisciplinary panel with members from neurosurgical, anesthesiological, geriatric and neuroradiological societies, and based on a literature search including studies published up until March 2024, consensus was achieved on the role of MMAE in CSDH management. The consensus statement acknowledges three recent randomized trials on MMAE (EMBOLISE, STEM and MAGIC-MT), but without full results analysis, since these studies were not yet published at the time of the consensus review. However, almost synchronously to the publication by Bartek J Jr *et al*, these trials were published in full (Liu et al., 2024; Davies et al., 2024; Fiorella et al., 2024). The expected impact of EMBOLISE, STEM and MAGIC-MT on CSDH management is significant and therefore the consensus statement by Bartek J *et al,* though recently published, needs further consideration.

The international Collaborative Research Initiative on Chronic subdural hematoma (iCORIC) was established in 2018 by research-active clinicians within the field of CSDH and is supported by the EANS (European Association of Neurosurgical Societies). The purpose of this international collaborative was to create a forum to encourage and share high-quality research and clinical guidelines within this field (Edlmann et al., 2020). The collaborative committee meets annually and has recently completed an international Delphi Survey and consensus meeting for a core outcome set in CSDH pending imminent publication (Holl et al., 2021). Bartek J is a member of iCORIC, but not included as an author of this letter.

The authors of this letter, the iCORIC steering committee, recognizes the role of MMAE in the three scenarios suggested by Bartek J *et al*; MMAE as a “stand-alone” treatment in *de novo* or recurrent symptomatic CSDH in which surgery is prevented as suspension of anti-thrombotics may lead to an unacceptable high risk of thromboembolic events, and as an adjunct to surgery in cases of recurrent CSDH (Bartek et al., 2024). Although, with such large numbers of patients on anti-thrombotics, at which point their thromboembolic event rate from suspension becomes unacceptable is open to broad interpretation.

In EMBOLISE, STEM and MAGIC-MT, MMAE as a treatment option in recurrent CSDH was not investigated, and high-quality evidence for the last statement is still lacking. However, a response from Bartek J *et al* on the importance of the EMBOLISE, STEM and MAGIC-MT as either a stand-alone or an adjunct in *de novo* symptomatic CSDH is warranted. The following section discusses iCORIC's reflections on the newly published trials.

The EMBOLISE trial (Davies et al., 2024) randomised patients with symptomatic CSDH to burr holes (BH) alone versus BH + MMAE. The results showed significantly lower re-operation rate in the BH + MMAE group compared to BHs alone (4.1% versus 11.3%). This trial is a good replication of clinical practice, with recurrence rates in the BH group comparable to the literature, however it has a number-needed-to-treat (NNT) of 14 patients. From both a logistic and health economical perspective, this number is likely to be too high to consider MMAE as a standard treatment to all patients with *de novo* CSDH to prevent a small number of recurrences. Furthermore, despite the increased intervention rate the functional outcomes and mortality did not significantly differ, questioning how much this intervention improves the quality of patients’ lives.

The STEM trial (Fiorella et al., 2024) investigates MMAE as an adjunct to standard treatment in two groups; surgically treated CSDH and non-surgically treated CSDH. The primary outcome was a composite of radiological recurrence, re-operation, thromboembolic complications, or death from a neurological cause. Adjunctive MMAE resulted in a significant lower risk of treatment failure without increasing thromboembolic complication. However, the effect was primarily driven by the non-surgically treated group, a sub-group with uncertainty as to whether treatment is required at all, given the potential for spontaneous resolution. Furthermore, a NNT was not presented. Whilst these results suggest that early MMAE *may* prevent CSDH progression in some patients, this data is not sufficient to determine this as a standard treatment for patients with CSDH which would not normally be operated on. Also, the reported recurrence rate for BH treated patients was 30%, which is notably higher compared to previously reported data, and 10% for MMAE treated patients, which corresponds better with the average recurrence rate for BH treated patients in modern series (Hutchinson et al., 2020; Soleman et al., 2019; Jensen et al., 2021; Miah et al., 2023; Duerinck et al., 2022).

The MAGIC MT trial (Liu et al., 2024) investigates MMAE versus standard care in patients with symptomatic CSDH, where the majority (78%) received BHs. There was a significant increase in serious adverse events in the standard-care arm, which was largely accounted for by a small increase in deaths (not procedure related) and a non-significant increase in re-operation rates, nearly 2/3 of which were in the group of patients managed non-surgically initially. Only 14% of the group managed conservatively ever went on to have surgery, suggesting that the majority of these patients never needed treatment and that the MMAE may have reduced surgical intervention in some patients but might not have been necessary in many cases. It is noticeable that overall recurrence rates were very low (4.7–5.2%) compared to the literature, questioning the generalizability of the trial (Hutchinson et al., 2020; Soleman et al., 2019; Jensen et al., 2021; Miah et al., 2023; Duerinck et al., 2022; Iorio et al., 2016).

When considering the benefit, or non-inferiority of MMAE, it is important to consider the wider picture within each healthcare setting. Whilst MMAE may be thought as “less invasive”, as Bartek J *et al* discuss, this procedure is often done under general anesthesia (where BHs most often are not), can take a considerable length of time (particularly in less experienced facilities), and requires significant financial and logistical changes to set-up a new service. If BHs can already be readily accommodated by a widely available set of skilled clinicians, potentially in a shorter time frame and at a lower cost, then this may still be preferrable in the majority of cases. To the knowledge of iCORIC, BH alone remains economically more favorable than BH with adjacent MMAE, and therefore a change in practice would need to prove a rather low NNT. Very little is discussed about patient perceptions and experience in relation to these procedures, as well as their quality of life, areas which needs more in-depth research to understand how comparable they are.

In summary, we consider that the recently published MMAE trials (EMBOLISE, STEM, MAGIC-MT) have shown promising effects in CSDH. However, with widely reported good outcomes and low recurrence rates (_∼_10%) with modern surgical treatment, careful consideration is needed about the application of this new intervention. Widespread adoption for all patients is not appropriate, and we support the consensus statement in its current form, selecting patients carefully. One must be careful not to apply an effective, yet expensive, solution to a problem most patients do not have.

## Declaration of competing interest

The authors declare that they have no known competing financial interests or personal relationships that could have appeared to influence the work reported in this paper.
